# Cardiomyocyte protective effects of thyroid hormone during hypoxia/reoxygenation injury through activating of IGF‐1‐mediated PI3K/Akt signalling

**DOI:** 10.1111/jcmm.16389

**Published:** 2021-03-16

**Authors:** Bin Zeng, Lei Liu, Xiaoting Liao, Caixia Zhang

**Affiliations:** ^1^ Hubei Key Laboratory of Cardiology Department of Cardiology Cardiovascular Research Institute Renmin Hospital of Wuhan University Wuhan University Wuhan China; ^2^ Department of Cardiology Hubei No. 3 People’s Hospital of Jianghan University Wuhan China

**Keywords:** Akt signalling pathway, apoptosis, Ca^2+^ homeostasis, mitochondrial membrane potential, PI3K, triiodothyronine

## Abstract

Ischaemia/reperfusion (I/R) injury is a common clinical condition that results in apoptosis and oxidative stress injury. Thyroid hormone was previously reported to elicit cardiac myocyte hypertrophy and promote cardiac function after cardiac injury. We used an in vivo mouse model of I/R injury and in vitro primary cardiomyocyte culture assays to investigate the effects of thyroid hormone on cardiomyocytes during hypoxia/reoxygenation (H/R) injury. The results showed that T3 pretreatment in vivo significantly improved left ventricular function after I/R injury. In vitro, T3 pretreatment decreased cell apoptosis rate, inhibited caspase‐3 activity and decreased the Bax/Bcl‐2 ration induced by H/R injury. T3 pretreatment significantly attenuated the loss of mitochondrial membrane potential. Furthermore, it was observed that T3 diminished the expression of NCX1 protein and decreased SERCA2a protein expression in H/R‐induced cardiomyocytes, and T3 prevented intracellular Ca^2+^ increase during H/R injury. Also, T3 increased the expression of IGF‐1, and PI3K/Akt signalling in cardiomyocytes under H/R‐induced injury, and that the protective effect of T3 against H/R‐induced injury was blocked by the PI3K inhibitor LY294002. IGF‐1 receptor (IGF‐1R) inhibitor GSK1904529A significantly inhibited the expression of IGF‐1R and PI3K/Akt signalling. In summary, T3 pretreatment protects cardiomyocytes against H/R‐induced injury by activating the IGF‐1‐mediated PI3K/Akt signalling pathway.

## 1 INTRODUCTION

Ischaemia/reperfusion (I/R) injury is a common clinical manifestation of ischaemic heart disease,^1^ which can cause myocardial inflammation and apoptosis, resulting in irreversible damage and dysfunction in the heart.^1^, ^2^, ^3^ In the process of reperfusion, excessive reactive oxygen species (ROS) levels facilitate and lead to the opening of the mitochondrial permeability transition pore (mPTP), which induces mitochondrial depolarization and aggravates cell apoptosis.^4^, ^5^ Furthermore, large amounts of ROS generated during I/R injury could lead to impaired intracellular Ca^2+^ homeostasis and trigger cell death.^6^, ^7^ These numerous pathological processes aggravate heart injury, which results in arrhythmia, microvascular obstruction and eventually heart failure. Thus, preventing cardiomyocyte loss and reversing cardiac damage in the early stages are important to repair cardiac injury and improve prognosis.

It has been recognized that thyroid hormone (TH) has important functions in the regulation of cardiac mechanical function and electrophysiological properties. Severe heart diseases, including acute myocardial infarction (AMI) and heart failure, are often accompanied by altered metabolism of THs or TH receptors (TRs).^8^, ^9^ TH is considered a major regulator of cardiovascular function, including regulation of contractile and calcium‐handling protein expression, ion channels and sympathetic tonus in cardiovascular system homeostasis.^10^, ^11^ Disruption of thyroid system homeostasis could lead to severe alterations in the biochemistry, structure and contractility of cardiac muscle.^12^ Previous studies have reported that low‐triiodothyronine (T3) treatment after AMI could promote cardiac function and prevent cardiac remodelling,^8^, ^13^ and that the enhanced myocyte contractility is related to an increase in the L‐type Ca^2+^ channel current.^13^

Insulin‐like growth factor (IGF)‐1 acts as an essential regulator of cardiac structure and function, such as inhibition of apoptosis, promotion of cell growth and augmentation of calcium signalling.^14^ IGF‐1 could effectively improve cardiac function recovery after myocardial infarction (MI), and the protective effect of IGF‐1 on cell survival was mediated by activating various signalling pathways, including the PI3K/Akt signalling pathway.^15^ PI3K plays a critical role in controlling the balance between cell survival and apoptosis, and Akt is the downstream target of PI3K.^15^, ^16^ Activated Akt can play a central role in preventing cell apoptosis, including modulating the activities of the Bcl‐2 family, caspase‐9 and caspase‐3.^16^ Moreover, activated Akt could maintain mitochondrial function and regulate different ion channels in myocytes.^17^, ^18^ Previous studies have reported that TH could activate Akt signalling in cardiomyocytes,^19^, ^20^ and whether activation of Akt signalling is due to a direct effect of thyroid hormone during ischaemia/reperfusion injury is still unknown.

In the present study, we aimed to evaluate whether pretreatment with T3 can improve cardiomyocyte survival and cell function after hypoxia/reoxygenation (H/R) injury. In addition, we sought to determine whether the protective effect from T3 pretreatment was facilitated by the IGF‐1‐mediated PI3K/Akt signalling pathway.

## 2 MATERIALS AND METHODS

### 2.1 Animal I/R model and study protocols

Male C57BL/6 mice aged 8‐10 weeks (weight: 24‐29 g) were purchased from Beijing Vital River Laboratory Animal Technology Co., Ltd, (Beijing, China). The animal protocols in this experiment were approved by the Animal Care and Use Committee of Renmin Hospital of Wuhan University and conformed to the Guidelines for the Care and Use of Laboratory Animals published by the US National Institutes of Health. Mice were anaesthetized with 3% sodium pentobarbital at a dose of 80 mg/kg (in traperitoneally). A left thoracotomy was performed at the third or fourth intercostal space, and the left anterior descending coronary artery (LAD) was ligated with slipknot (6‐0 silk). The slipknot was released after 45 minutes of ischaemia following a 2 hours of reperfusion.

Mice were randomly divided into 5 groups: (1) Sham group, silk was placed underneath the LAD without ligated; (2) I/R group, LAD was ligated for 45 min and received 2 hours of reperfusion using a vehicle (0.9% NaCl, intravenously); (3) I/R + T3 group, received an injection of T3 at the dose of 1.4 μg/100 g/d; (4) I/R + T3+LY group, received an injection of T3(1.4 μg/100 g/d) and LY294002 (3 mg/100 g/d); (5) I/R + LY group, received an injection of LY294002 (3 mg/100 g/d). Drugs or vehicle was administered intraperitoneally daily for 3 days before surgery, each mouse received the same total dose every day by adjusting the dose of saline.

### 2.2 Echocardiography

Cardiac echocardiography was performed for the experimental mice at 7th day post‐operation. Mice in each group (n = 6) were anaesthetized with 1.5%‐2% isoflurane by inhalation, and echocardiography was then performed using a MyLab30CV ultrasound (Biosound Esaote Inc Indianapolis) with a 12.5‐MHz linear array ultrasound transducer. The left ventricular ejection fraction (LVEF), fractional shortening (FS), the left ventricular end‐diastolic diameter (LVEDd) and left ventricular end‐systolic diameter (LVESd) were measured from M‐mode recording.

### 2.3 Primary cardiomyocyte culture and induction of H/R model in vitro

Animals were purchased from the animal centre of Hubei Province, and animal experiments were performed in accordance with the Guide for the Care and Use of Laboratory Animals published by the US National Institutes of Health (NIH Publication No. 85‐23, revised 1996) and approved by the Animal Care and Use Committee of Renmin Hospital of Wuhan University (protocol number: 00013518). Primary cultures of neonatal mouse cardiomyocytes were prepared by enzymatic disaggregation of cardiac tissue obtained from 1‐ to 2‐day‐old C57bl/6 mice, as described previously.^29^ Briefly, hearts were obtained and immersed in 0.25% trypsin (cat. no. C0201‐100 mL; Beyotime, Shanghai, China) at 4°C overnight. The hearts were digested with type II collagenase (cat. no.9001‐12‐1; Sigma‐Aidrich) at 37°C, and the isolated cells were filtered and centrifuged at 1000 rpm for 10 minutes. Then, the cells were cultured for 90 minutes at 37°C for cardiomyocyte purification, and the cardiomyocytes were finally cultured at a concentration of 1 × 10^5^ cells/ml in medium containing 10% heat inactivated foetal bovine serum (FBS; cat. no.16140071; Gibco), 1% penicillin‐streptomycin (cat. no. C0222; Beyotime) and 5‐bromo‐2‐deoxyuridine (1:100; cat. no. 0000059143; Sigma‐Aidrich) to inhibit fibroblast growth. The cells were incubated at 37°C in a humidified incubator with 5% CO_2_ for 48 hours prior to treatment.

For experiment under hypoxia/reoxygenation condition, cardiomyocytes media were replaced with phosphate buffer saline (PBS; cat. no. C0221A; Beyotime) prior to incubation in a tri‐gas incubator (95% N_2_, 5% CO_2_) at 37°C for 4 hours, then the PBS was replaced with the culture medium containing 10% FBS and cultured in a standard incubator (95% O_2_, 5%CO_2_) at 37°C for 24 hours.

For T3 pretreatment, 10, 20, 40 and 80 μmol/L T3 (cat. no.ab30804; Abcam) were added into the culture media for 24 hours prior to H/R. 2‐(4‐morpholinyl)‐8‐phenyl‐chromone (LY294002; cat. no. HY‐10108; MCE) is a specific inhibitor of PI3K/Akt signalling,^8^ was added into culture medium for 24 hours prior to H/R conditions, at a final concentration of 20 μmol/L. IGF‐1R inhibitor GSK1904529A (cat. no.1089283‐49‐7; MCE, USA, 20 nmol/L) was added to the culture medium for 4 hours prior to T3 pretreatment to block IGF‐1 activation.

To explore the protective mechanisms of T3 pretreatment against cardiomyocyte H/R injury, cultured cardiomyocytes were randomly divided into five groups: control group, H/R group, H/R + T3 group, H/R + T3 + LY group and H/R + LY group.

### 2.4 Cell viability assay

Cell viability was measured using a CCK‐8 detection kit (cat. no. C0038; Beyotime). Cardiomyocytes were plated in a 96‐well plate (5000 cells/well). After adhering overnight, cells were pretreatment with 10, 20, 40 and 80 μmol/L T3 for 24 hours prior to H/R injury, respectively. Then, 10 μL CCK‐8 was added into the culture medium, and the cells were incubated for 4 hours at 37℃. The absorbance of each culture well was measured by using Microplate Reader (TECAN infinite M200 PRO) at a wavelength of 450 nm. The cell viability rate was expressed as a percentage of absorbance compared to the control.

### 2.5 Apoptosis detection

Apoptosis was measured using an Annexin V‐FITC/PI apoptosis detection kit (BD Biosciences) according to the manufacturer's protocol. Briefly, after H/R treatment, the cardiomyocytes were washed twice with cold PBS, then the cells were collected and suspended in 1× annexin V binding buffer at a concentration of 10^6^/mL, 100 μL (10^5^ cells) was placed in a 5 mL tube followed by the addition of 5 μL Annexin V‐FITC, the cells were gently mixed and incubated for 15 minutes at room temperature in the dark. Then 5 μL PI was added into the cells and gently mixed followed by 5 minutes incubation at room temperature in the dark. The samples were immediately analysed using a flow cytometer (Becton Dickinson). Approximately 20 000 cardiomyocytes were counted for each sample, and data were analysed with the Expo32 software.

### 2.6 Western blotting

After H/R treatment, cells were harvested immediately and lysed in 1× RIPA buffer (cat. no. AS1004; ASPEN) at 4°C. The lysates were centrifuged, and the protein concentration was determined with a bicinchoninic acid (BCA) protein assay kit (cat. no. As1086; ASPEN). Protein samples were separated by 12% SDS‐PAGE electrophoresis and then transferred to PVDF membranes. The membranes were first blocked in 5% non‐fat milk for 30 minutes and then incubated overnight at 4°C with primary antibodies against PI3K (dilution: 1:5000; 4292, CST), Akt (dilution: 1:5000; 4691, CST), IGF‐1 (dilution: 1:5000; DF6096, Affbiotech) and IGF‐1R (dilution: 1:5000; ab39675, Abcam), caspase 3 (dilution: 1:1000; ab49822, Abcam), Bax (dilution: 1:1000; 2772, CST), Bcl‐2 (dilution: 1:1000; ab59348, Abcam), SERCA2a (dilution: 1:1000; ab3625, Abcam) and NCX1 (dilution: 1:1000; ab177952, Abcam). Then, the membranes were washed 3 times in 1× Tris‐buffered saline tween (TBST) buffer and incubated with horseradish peroxidase (HRP)‐conjugated goat anti‐rabbit secondary antibodies (dilution: 1:10 000; AS1107, ASPEN) for 90 minutes at room temperature. The protein bands were detected with an enhanced chemiluminescence (ECL) system (ASPEN, Australia), visualized with the Chemi Doc XRS+ gel documentation system and analysed using AlphaEaseFC software. The expression levels of β‐actin served as an internal control for protein loading to further assure the same volume for all the samples.

### 2.7 Measurement of mitochondrial membrane potential (∆Ym)

The change in mitochondrial membrane potential was detected by JC‐1 staining. After H/R injury, cardiomyocytes were stained with JC‐1 (cat. no. M8650; Solarbio) at a final concentration of 2 μmol/L in dark at 37°C for 30 minutes. Then the cells were washed three times with PBS, and images with JC‐1 staining were observed under a high‐content imaging system Image Xpress Micro (Molecular Devices). For further analysis of fluorescence intensity, a microplate reader (TECAN Infinite Mi000) was used to analyse the changes. The green JC‐1 signal was detected at excitation wavelengths of 514 nm and emission wavelengths of 529 nm, the red signal was detected at excitation wavelengths of 585 nm and emission wavelengths of 590 nm.

#### 2.7.1 Intracellular Ca^2+^ measurement

Acetoxymethyl ester of Fluo3 (Fluo‐3/AM) is widely used for intracellular Ca^2+^ detection. After H/R treatment, the cardiomyocytes were washed with PBS, and then cells were incubated with 5 µmol/L Fluo‐3/AM (cat. no. S1056; Beyotime) for 1 hour at 37°C in dark. After loading, the cells were incubated for 20 minutes at room temperature to ensure the complete transformation of fluo‐3 AM into fluo‐3. The intracellular Ca^2+^ levels were measured at an excitation wavelength of 488 nm and an emission wavelength of 525 nm by using flow cytometer (Becton Dickinson). Approximately 20 000 cardiomyocytes were counted for each sample, and data were analysed with the Expo32 software.

### 2.8 Data analysis

The results are presented as the mean ± SD. All current amplitudes were normalized to cell capacitance and expressed as pA/pF. Student's unpaired t test was used to compare 2 independent samples, and one‐way ANOVA was used to compare different data from multiple groups with SPSS 22.0. Nonlinear curve fitting was performed using Origin 8 software. Values of *P* < .05 were considered significant.

## 3 RESULTS

### 3.1 T3 pretreatment improved cardiac function after I/R injury in vivo

**FIGURE 1:**
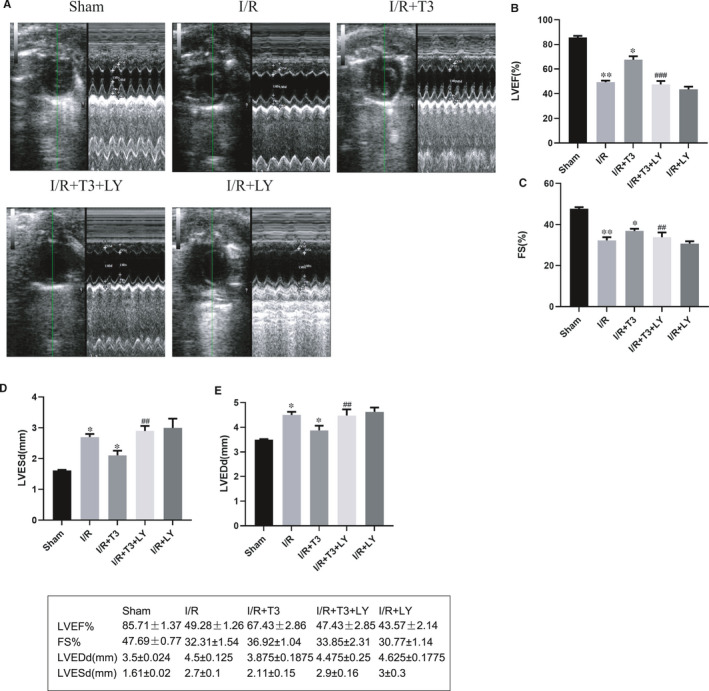
T3 improved cardiac function after I/R injury. A, Left ventricular mode echocardiograms in short axis at 7th day after operation. B, Left ventricular ejection fraction (%LVEF). C, Fractional shortening (%FS). D, Left ventricular end‐systolic diameter (LVESd). E, Left ventricular end‐diastolic diameter (LVEDd). The values were expressed as the mean ± SD in the experiment. n = 6. ·*P* < .05 vs Sham, ··*P* < .01 vs Sham, ····*P* < .0001 vs Sham; ^*^ *P* < .05 vs I/R, ^**^ *P* < .01 vs I/R, ^****^ *P* < .0001 vs I/R; ^##^ *P* < .01 vs I/R + T3, ^###^ *P* < .001 vs I/R + T3

Echocardiography was conducted to assess global systolic and diastolic function in mice after I/R injury. The results indicated that left ventricular function was impaired after I/R injury, which were characterized by decreased LVEF and FS and increased LVESd and LVEDd values (*P* < .05). However, T3 pretreatment obviously restored cardiac function, as evidenced by preserved LVEF and FS and reduced LVESd and LVEDd values (*P* < .05). In contrast, LY294002 suppressed the effects of T3 stimuli and aggravated I/R‐induced cardiac dysfunction (*P* < .05) (Figure 1).

### 3.2 Protective effects of T3 on H/R‐induced apoptosis in neonatal mouse cardiomyocytes

**FIGURE 2:**
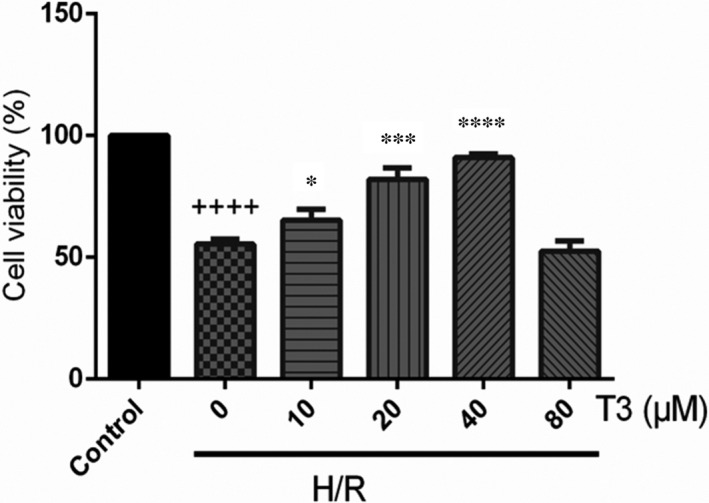
T3 increased cell viability of cardiomyocytes under H/R‐induced injury. The CCK‐8 assay was performed to examine the cell viability. ^++++^ *P* < .0001 vs control, ^*^ *P* < .05 vs H/R, ^***^ *P* < .001 vs H/R, ^****^ *P* <.0001 vs H/R

As shown in Figure 2, compared with control group, the viability of cardiomyocytes was markedly decreased to 55.42% after H/R‐induced injury (n = 3, *P* < .0001). However, pretreatment with T3 increased cell viability under H/R‐induced injury. Pretreatment with 10, 20 and 40 μmol/L T3 for 24 hours significantly increased the cell viability to 65.28% (n = 3, *P* < .05), 82.09% (n = 3, *P* < .001), and 90.97% (n = 3, *P* < .0001), respectively, of that of control cardiomyocytes (Figure 4A). Moreover, pretreatment with 80 μmol/L T3 reduced cell viability to 52.5%. Thus, we used 40 μmol/L T3 as an optimal concentration for the following study.

**FIGURE 3:**
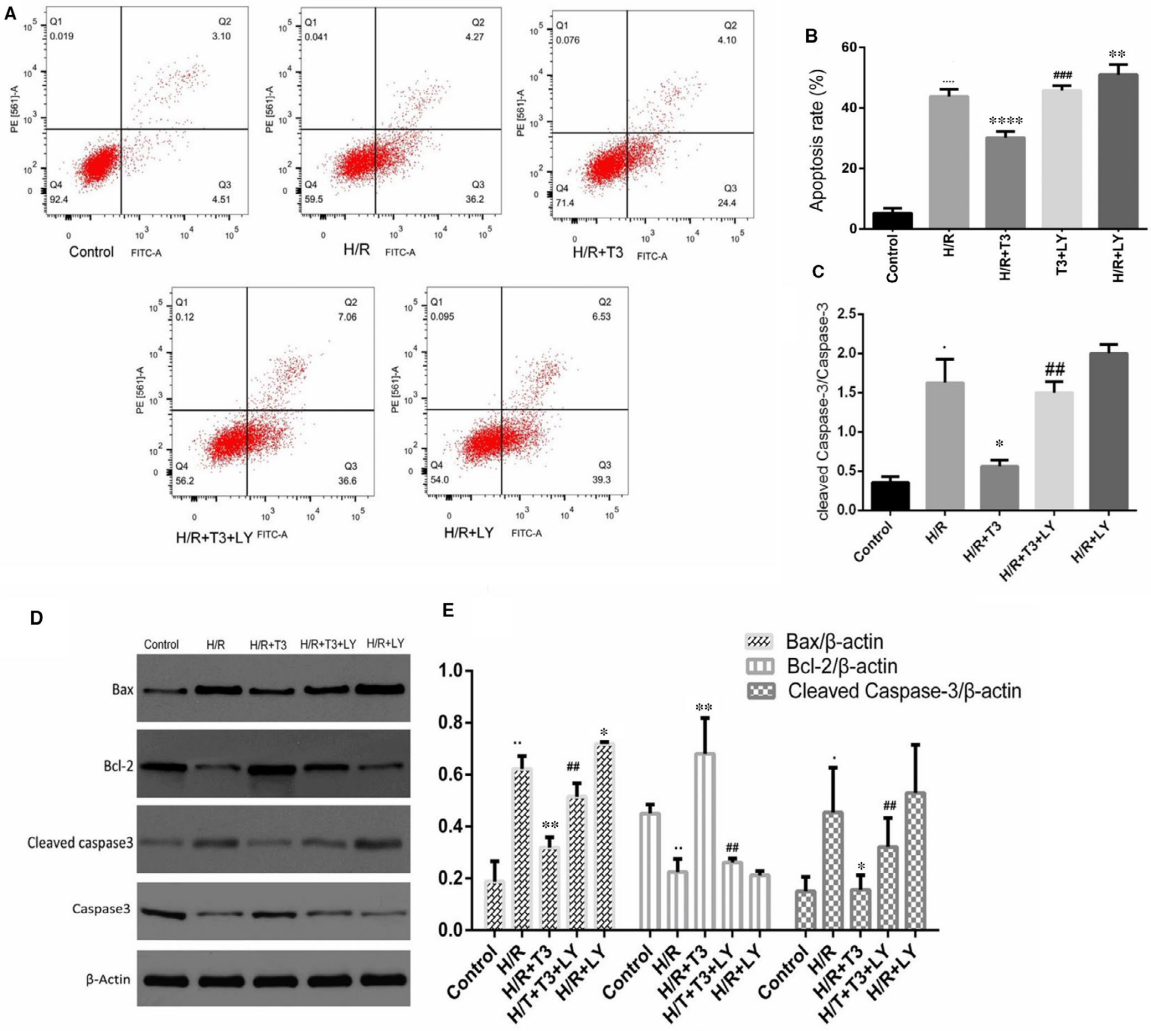
T3 protected cardiomyocytes from H/R‐induced apoptosis. A, Apoptosis in cardiomyocytes was analysed by flow cytometry and (B) Quantitative analysis of the percentages of apoptotic cells in each group. C, Data acquired from Western blotting showed the ratio of cleaved capase‐3/caspase‐3 in each group. D, The expression of Bax, Bcl‐2 and cleaved caspase‐3 was analysed by Western blotting and E, Representative bands quantified in the corresponding bar graph. β‐actin protein expression was used for normalization. The values were expressed as the mean ± SD in the experiment. n = 6. ·*P* <.05 vs control, ··*P* <.01 vs control, ····*P* <.0001 vs control; ^*^ *P* <.05 vs H/R, ^**^ *P* <.01 vs H/R, ^****^ *P* <.0001 vs H/R; ^##^ *P* <.01 vs H/R + T3, ^###^ *P* <.001 vs H/R + T3

As shown in Figure 3, H/R treatment induced a high rate of cardiomyocyte apoptosis, with the apoptosis rate increased from 5.32% ± 0.6% to 42.1% ± 2.1% (n = 3, *P* < .0001); however, T3 decreased the number of annexin V‐positive/PI‐negative and Annexin V‐positive/PI‐positive cells, and the apoptosis rate decreased to 24.47% ± 1.6%, suggesting that T3 inhibited H/R‐induced cell apoptosis both in the early and late stages. LY294002, a PI3K inhibitor, was used to inhibit the phosphorylation of the PI3K/Akt pathway. In the present study, LY294002 significantly diminished the effect of T3 on H/R‐induced cell apoptosis, resulting in an increased apoptosis rate (n = 3, *P* < .05).

After H/R‐induced injury, as shown in Figure 3, a pronounced increase in the expression of Bax and cleaved caspase‐3 was found in H/R group, with an increased expression level of 3.31‐fold (n = 3, *P* < .01) and 3.01‐fold (n = 3, *P* < .05), respectively, compared with that of control group. The Bcl‐2 protein expression was decreased to 0.491‐fold (n = 3, *P* < .01) in H/R‐induced cardiomyocytes. However, T3 pretreatment significantly decreased the Bax and cleaved caspase‐3 expression levels to 1.71‐fold and 1.04‐fold (n = 3, *P* < .05 and *P* < .01), and the Bcl‐2 expression was increased to 1.51‐fold (n = 3, *P* < .01). LY294002 markedly inhibited the T3‐mediated decrease in Bax and cleaved caspase‐3 expression, and decreased the Bcl‐2 expression (n = 3, *P* < .01).

### 3.3 T3 prevented mitochondrial membrane potential changes in cardiomyocytes during H/R injury

**FIGURE 4:**
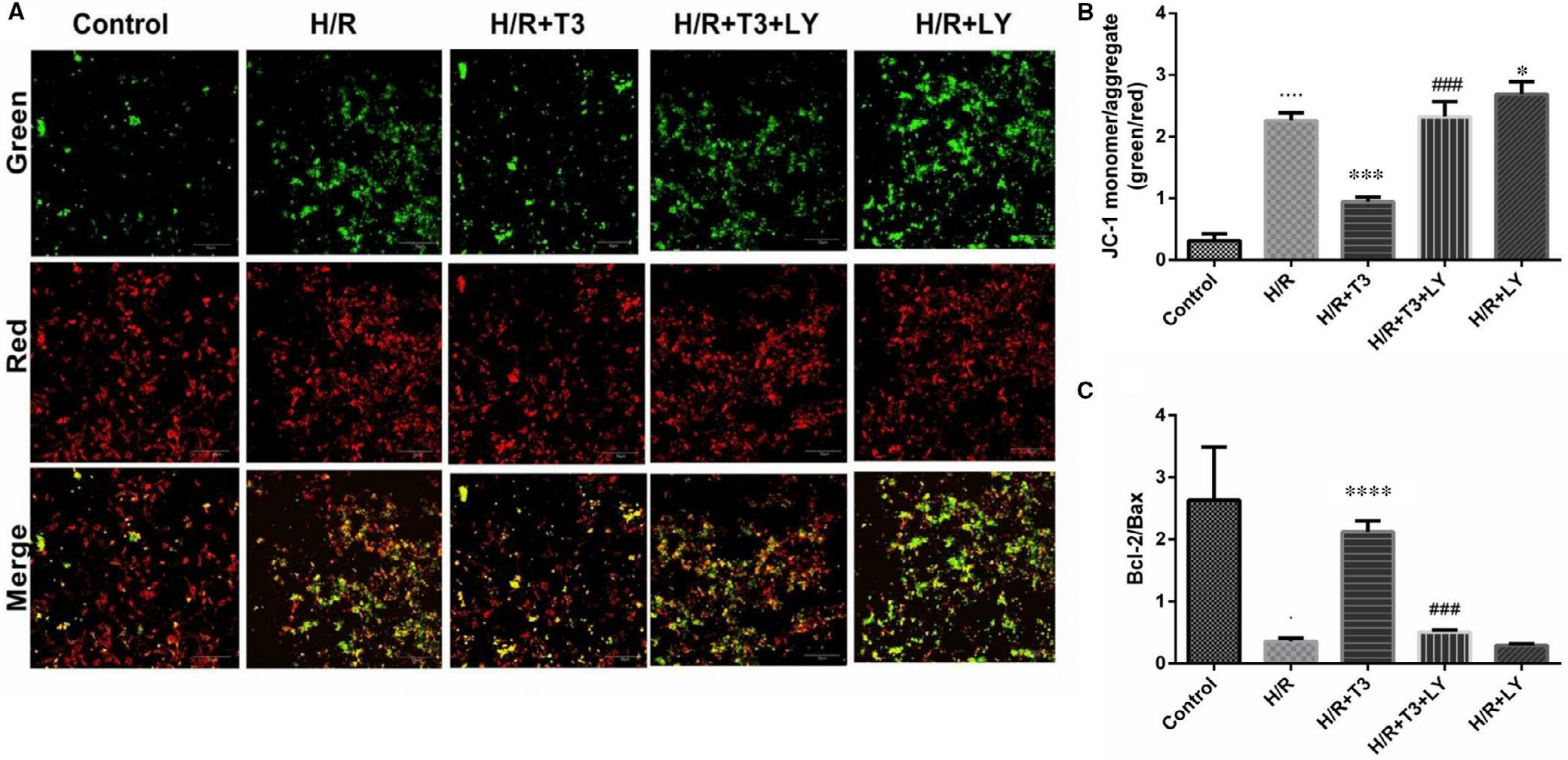
The effect of T3 on H/R‐induced depolarization of mitochondrial membrane in neonatal ventricular cardiomyocytes. A, Representative images of JC‐1 green/red and merges in five different groups. B, The bar diagram showing the ratio of green to red fluorescence intensity in five different groups. C, The bar diagram showing the ratio of Bcl‐2/Bax protein expression. The values were expressed as the mean ± SD in the experiments. n = 6. ····*P* <.0001 vs control;^***^ *P* <.001 vs H/R; ^###^ *P* <.001 vs H/R + T3; ^+^ *P* <.05 vs H/R ·*P* <.05, ····*P* <.0001 vs control; ^*^ *P* <.05, ^***^ *P* <.001, ^****^ *P* <.0001 vs H/R; ^###^ *P* <.001 vs H/R + T3

Mitochondria play a critical role in H/R injury, and disruption of the mitochondrial membrane potential is an early event in the cell apoptotic process. As shown in Figure 4A,B, H/R treatment increased the ratio of green to red fluorescence among cardiomyocyte mitochondria from 0.35 ± 0.069 to 2.18 ± 0.087 (n = 3, *P* < .0001), indicating that H/R injury decreased the mitochondrial membrane potential of cardiomyocytes. However, this ratio was significantly decreased to 0.85 ± 0.18 with T3 pretreatment (n = 3, *P* < .001), indicating that T3 could attenuate loss of mitochondrial membrane potential induced by H/R injury. This effect of T3 was markedly abolished by LY294002 pretreatment, resulting in a significantly increased green to red fluorescence ratio to 2.15 ± 0.12 in cardiomyocytes (n = 3, *P* < .001).

During H/R injury, the ratio of Bcl‐2/Bax protein expression was significantly decreased from 2.63 ± 0.49 to 0.36 ± 0.03 (n = 3, *P* < .05); T3 pretreatment significant reversed this effect, resulting a marked increase in the Bcl‐2/Bax ratio to 2.12 ± 0.11 (n = 3, *P* < .0001). However, LY294002 diminished this effect of T3 pretreatment and significantly increased the Bcl‐2/Bax ratio to 0.39 ± 0.07 (n = 3, *P* < .001) (Figure 4C).

### 3.4 T3 regulated the expression of proteins involved in Ca^2+^ homeostasis in cardiomyocytes

**FIGURE 5:**
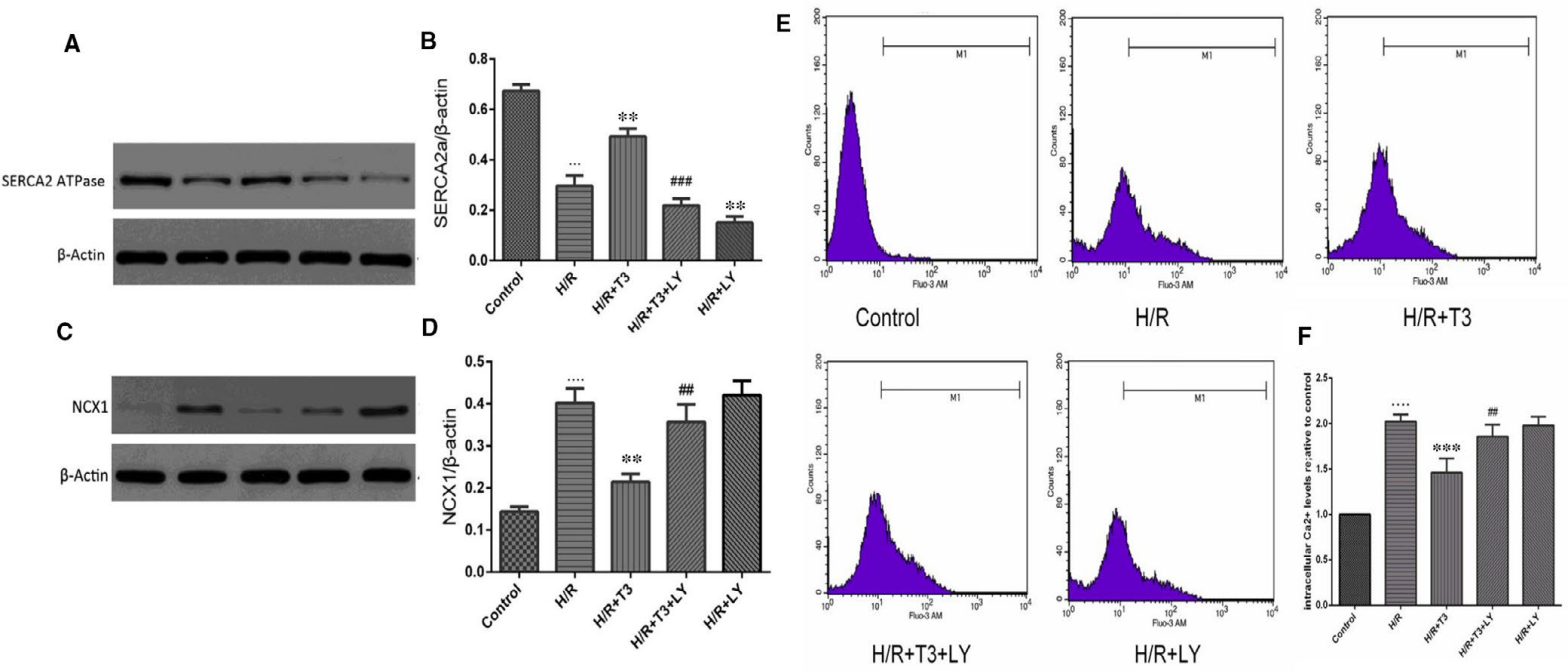
T3 ameliorated intracellular Ca^2+^ overload and prevented the intracellular Ca^2+^ increase in cardiomyocyte during H/R injury. A, The expression of SERCA2a protein expression in neonatal mouse cardiomyocytes; β‐actin was used for normalization. B, Representative bands quantified in the corresponding bar graph. C, The expression of NCX1 protein expression in neonatal mouse cardiomyocytes; β‐actin was used for normalization. D, Representative bands quantified in the corresponding bar graph. (E and F) Cardiomyocytes were stained individually by Fluo‐3/AM dye for intracellular Ca^2+^. The results were determined with flow cytometry and fluorescence intensity of control in each group. The values were expressed as the mean ± SD three independent experiments. n = 6. ···*P* <.001, ····*P* <.0001 vs control; ^**^ *P* <.01 vs H/R; ^***^ *P* <.001 vs H/R; ^##^ *P* <.01 vs H/R + T3, ^###^ *P* <.001 vs H/R + T3

As shown in Figure 5A,B, compared to that in the control group, SERCA2a expression was sharply decreased in H/R‐induced cardiomyocytes, and the reduction in expression was significantly reversed by T3 pretreatment (n = 3, *P* < .05). LY294002 significantly inhibited the T3‐induced increase in SERCA2a protein expression under H/R conditions.

The Na^+^–Ca^2+^ exchanger (NCX) plays a crucial role in maintaining Ca^2+^ homeostasis, but its reverse mode during ischaemia/reperfusion injury could exacerbate Ca^2+^ overload. As shown in Figure 4C,D, the level of Na^+^/Ca^2+^ exchanger (NCX1) protein expression in H/R‐induced cardiomyocytes was markedly increased, while T3 pretreatment significantly down‐regulated NCX1 protein expression in H/R injury (n = 3, *P* < .05). However, LY294002 significantly diminished T3‐induced decreases in NCX1 expression in cardiomyocytes under H/R conditions (n = 3, *P* < .05).

### 3.5 T3 prevented the intracellular Ca^2+^ increase induced by H/R injury

As shown in Figure 5E,F, compared to the control group, H/R injury significantly potentiated Fluo‐3 fluorescence; demonstrating an elevation in intracellular Ca^2+^ concentration, levels of intracellular Ca^2+^ were increased by 89% (n = 4, *P* < .0001). T3 pretreatment markedly inhibited this elevation of the intracellular Ca^2+^, and levels of intracellular Ca^2+^ in H/R + T3 group were decreased by 50.5% (n = 4, *P* < .001), compared with that in H/R group. However, the Fluo‐3 fluorescence in LY294002‐treated cardiomyocytes was increased, intracellular Ca^2+^ levels in H/R + T3 + LY group were increased by 70.08% (n = 4, *P* < .01), and in H/R + LY group, the levels were increased by 90.01%. The results indicated that T3 pretreatment could diminish H/R‐induced Ca^2+^ overload and maintain Ca^2+^ homeostasis.

### 3.6 IGF‐1 mediated PI3K/Akt signalling pathway accounts for the protective mechanism of T3 in H/R injury

**FIGURE 6:**
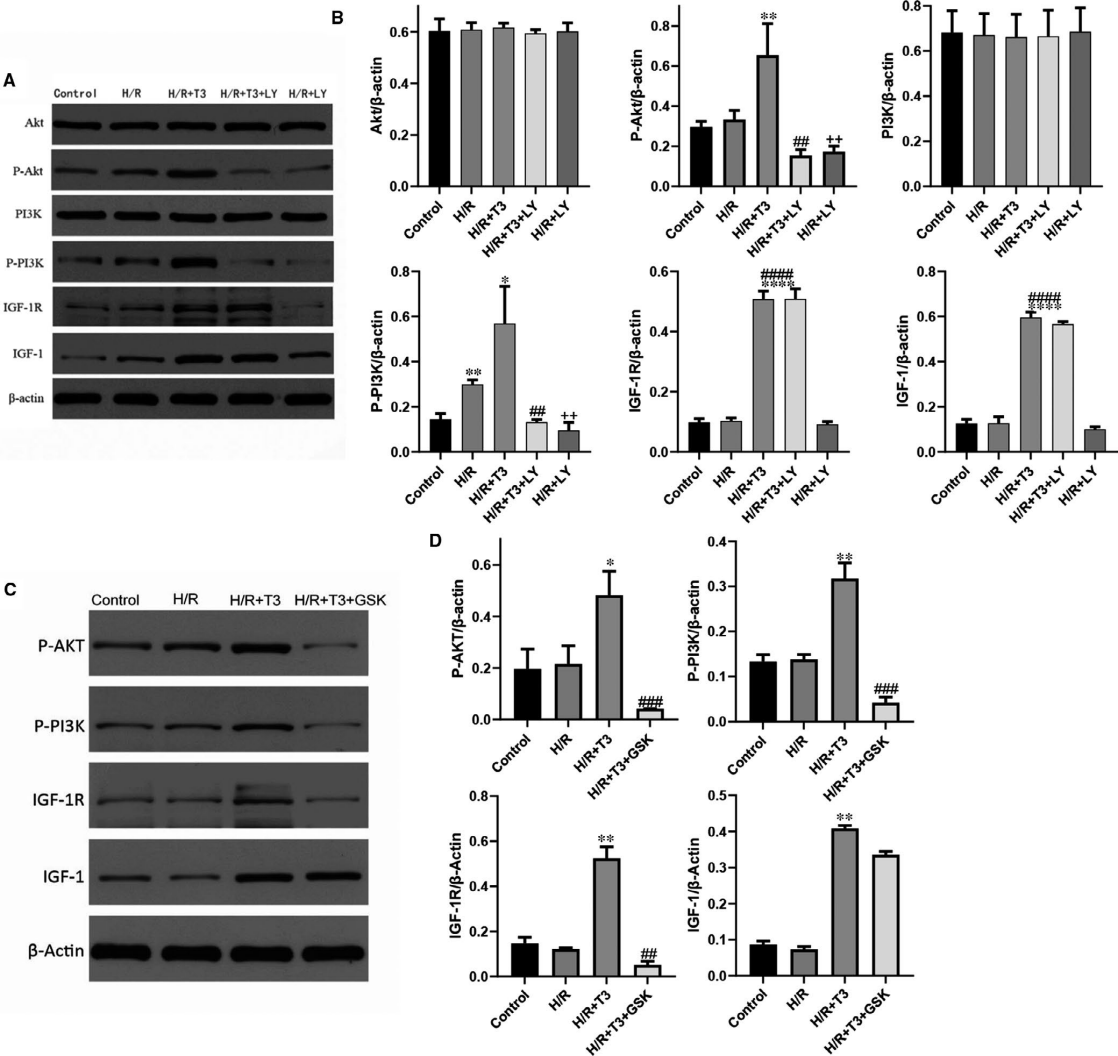
T3 protected cardiomyocytes from H/R‐induced apoptosis through PI3K/Akt signalling pathway (A and B). A, The expression of Akt, P‐Akt, PI3K, P‐Akt, IGF‐1, IGF‐1R; β‐actin protein expression was used for normalization and (B) representative bands quantified in the corresponding bar graph. The T3‐induced activation of PI3K/Akt signalling was mediated by IGF‐1/IGF‐1R (C and D). The expression of P‐Akt, P‐PI3K, IGF‐1 and IGF‐1R was analysed by Western blotting; and representative bands quantified in the corresponding bar graph. β‐actin protein expression was used for normalization. The values were expressed as the mean ± SD in the experiments. n = 6. ··*P* <.01 vs control; ^*^ *P* <.05, ^**^ *P* <.01, ^****^ *P* <.0001 vs H/R; ^##^ *P* <.01, ^###^ *P* <.001 vs H/R + T3

To investigate the underlying molecular mechanism of the effect of T3 on H/R‐induced cardiomyocytes, cells were pretreated with LY294002. As shown in Figure 6A,B, the results showed an increased p‐PI3K expression level in cardiomyocytes (n = 3, *P* < .05). T3 pretreatment significantly enhanced the expression of p‐P3K and p‐Akt (n = 3, *P* < .05 and *P* < .01). However, the phosphorylation of PI3K and Akt protein was significantly inhibited with LY294002 pretreatment (n = 3, *P* < .01). Furthermore, the protective effects of T3 on H/R‐induced cardiomyocytes were significantly inhibited, and the results were shown above. In addition, the results indicate that T3 pretreatment markedly increased the expression levels of IGF‐1 and IGF‐1R in H/R‐induced cardiomyocytes (n = 3, *P* < .0001), and this high increased levels of IGF‐1 and IGF‐1R proteins were not inhibited by LY294002 pretreatment.

To further confirm, the protective effects of T3 on H/R‐induced cardiomyocytes were mediated by IGF‐1, and cardiomyocytes were pretreated with IGF‐1R inhibitor GSK1904529A prior to T3 treatment. As shown in Figure 6C,D, T3 activated the PI3K/Akt signalling pathway, with a significantly increased P‐PI3K and P‐Akt proteins expression. However, the activation effect of T3 was inhibited by GSK1904529A, the results showed that the expression of IGF‐1R protein was decreased (n = 3, *P* < .01), and the phosphorylation of PI3K and Akt proteins was also significantly inhibited (n = 3, *P* < .001) in H/R + T3 + GSK1904529A group. These results indicate that the activation of PI3K/Akt signalling was through IGF‐1‐mediated. Thus, we have been suggested that T3 regulated the protective effect of cardiomyocytes in H/R‐induced injury through activating the IGF‐1‐mediated PI3K/Akt pathway.

## 4 DISCUSSION

Previous studies have demonstrated that T3 treatment after AMI could promote cardiac function and prevent cardiac remodelling.^8^, ^13^ Different from previous studies, this study analysed whether T3 pretreatment could protect cardiac function after I/R injury in vivo. As expected, echocardiographic evaluations showed obviously elevated LVEF and FS values and reduced LVESd and LVEDd values in T3 pretreatment mice, indicating that T3 pretreatment significantly improved left ventricular function in mice after I/R injury. Moreover, cardiomyocyte culture assays in vitro demonstrated that pre‐administration of T3 could reduce H/R‐induced cardiomyocyte apoptosis by preserving mitochondrial function and maintaining intracellular Ca^2+^ homeostasis. Furthermore, these biological effects were mediated via activation of the IGF‐1‐PI3k/Akt signalling pathway. Caspase‐3, the most critical apoptotic protease in the caspase cascade, was up‐regulated in H/R‐induced cardiomyocytes, which is accompanied by a significant increase in the cell apoptosis rate. T3 could alter the rate of cardiomyocyte apoptosis in an MI rat model.^13^ Our results also demonstrated the protective effect of T3 in cardiomyocytes, which involved the down‐regulation of caspase‐3 protein expression and a significant decrease in the cardiomyocyte apoptosis rate. Cardiomyocyte apoptosis is a main mechanism of myocardial I/R injury, and mitochondrial impairment is a leading cause of cell loss and contractile dysfunction in cardiac injury.^21^ During reperfusion injury, excessive ROS leads to the translocation of Bax protein to the outer mitochondrial membrane (OMM). A decreased ratio of Bcl‐2/Bax facilitated the formation of pores in mitochondria and induced mitochondrial depolarization and cytochrome c release, which induced the apoptotic pathway.^5^, ^21^, ^22^ Our results showed that H/R injury significantly decreased the Bcl‐2/Bax ratio; in addition, a reduced mitochondrial membrane potential was also found in H/R‐induced cardiomyocytes. However, these negative effects were significantly reversed with T3 pretreatment, which effectively inhibited H/R‐induced mitochondrial fission and induced a significant increase in mitochondrial membrane potential, thereby promoting the attenuation of H/R‐induced cell apoptosis. All these data suggested that T3 could protect cardiomyocytes by preventing cell apoptosis and promote the mitochondrial function of cardiomyocytes under H/R‐induced injury.

Ca^2+^ is a vital second messenger that plays several intracellular roles in the myocardium, including controlling cardiomyocyte mechanical behaviour, programmed cell death and cardiac muscle contraction.^23^ Ca^2+^ uptake and release are profoundly altered after cardiac ischaemia injury, resulting in impaired contractility and fatal arrhythmias. During ischaemia/reperfusion injury, down‐regulation of SERCA2a resulted in a decreased ability of Ca^2+^ intake in the sarcoplasmic reticulum.^22^ In addition, reperfusion injury increased the activation of NCX reverse mode, which eventually led to intracellular Ca^2+^ overload.^7^ A previous study revealed that the ratio of SRACA/phospholamban was obviously reduced in viable myocardium of AMI rat hearts and that long‐term T3 treatment after AMI could significantly increase this ratio and improve cardiac function.^24^ In the present study, our results also demonstrated that H/R injury significantly decreased SERCA2a expression and increased NCX1 protein expression levels in cardiomyocytes. Impaired Ca^2+^ homeostasis is a consequence of the dysregulation of Ca^2+^‐cycling proteins, and altered Ca^2+^ homeostasis resulted in abnormal ER stress and apoptosis.^25^ In the present results, H/R injury markedly increased intracellular Ca^2+^ levels in cardiomyocytes. However, pretreatment with T3 effectively inhibited intracellular Ca^2+^ increase in H/R‐induced cardiomyocytes and reversed the negative effect of H/R injury, which indicating that T3 might ameliorate Ca^2+^ reuptake and maintain Ca^2+^ homeostasis in H/R‐induced cardiomyocytes by mediating the transcriptional regulation of SERCA2a and NCX1 expression. Previous studies showed an enhanced contractile function of the left ventricle in an Akt overexpression mouse model with an enhanced amplitude of Ca^2+^ transients.^18^ Thus, we have been suggested that an alternative mechanism for the acute effect of T3 may occur through modulation of PI3k/Akt signalling pathway activity.

The PI3K/Akt signalling pathway is an important signal transduction pathway that plays a key role in cellular functions, including cell apoptosis, angiogenesis, autophagy and differentiation.^16^, ^17^ Activation of the PI3K/Akt signalling pathway could protect the heart against ischaemic injury by modulating the activities of Bcl‐2 family proteins and caspase‐3.^16^ Previous studies demonstrated a low basal level of the activation of PI3K and Akt in response to reperfusion/reoxygenation injury both in vivo & in vitro.^8^, ^16^ The present study also showed a low level of increased p‐PI3K in H/R‐induced cardiomyocytes. Both the induction of phosphor‐PI3K by H/R and the strongly enhanced phosphor‐PI3K and phosphor‐Akt by T3 were significantly blocked by LY294002. Moreover, the antiapoptotic effect of T3 was also abolished by LY294002. These results confirm the role of PI3K/Akt pathway in the T3 response on H/R‐induced cardiomyocytes.

IGF‐1 could effectively stimulate the PI3K pathway, producing phosphoinositides that promote Akt kinase activation.^15^ The present study showed an enhanced expression of IGF‐1 and IGF‐1R in the T3 pretreatment groups. Previous studies demonstrated the antiapoptotic and protective effect of IGF‐1 through activating of Akt signalling in different models of myocardial ischaemia as well as in isolated cardiomyocytes under hypoxia or oxidative stress.^15^, ^26^ GSK1904529A, a selective inhibitor for IGF‐1 receptor,^27^ could inhibited IGF‐1/PI3K/Akt signalling pathway.^28^ The present study showed that GSK1904529A inhibited the up‐regulation of IGF‐1R protein expression induced by T3 pretreatment. Moreover, the expression of phos‐PI3K and phos‐Akt protein was also suppressed by GSK1904529A. Thus, we have been suggested that activation of the PI3K/Akt signalling pathway was mediated by IGF‐1 and that the antiapoptotic effect of T3 was facilitated by the IGF‐1‐mediated PI3K/Akt signalling pathway.

In conclusions, we demonstrated that T3 protects cardiomyocytes against H/R‐induced injury by inhibiting cardiomyocyte apoptosis, attenuating the loss of cardiomyocyte mitochondrial membrane potential, and maintaining intracellular Ca^2+^ homeostasis in cardiomyocytes during H/R‐induced injury. Moreover, these effects are at least partially mediated by activating of IGF‐1/PI3K/Akt signalling pathway.

## CONFLICT OF INTEREST

On behalf of all authors, the corresponding author states that there is no conflict of interest.

## AUTHOR CONTRIBUTIONS

**Bin Zeng:** Funding acquisition (lead). **Lei Liu:** Writing‐original draft (lead). **Xiaoting Liao:** Methodology (equal); Writing‐review & editing (equal). **Caixia Zhang:** Methodology (equal); Writing‐review & editing (equal).
